# Analysis of related factors for sarco-osteoporosis in middle-aged and elderly inpatients and development and validation of a nomogram

**DOI:** 10.1186/s12891-023-06991-w

**Published:** 2024-03-28

**Authors:** Dao Juan Peng, Feng Qiong Gao, Yijiao Lou, Yan Ma, Tongxia Xia

**Affiliations:** 1 Nursing department, Affiliated Hospital of Zunyi Medical University, Dalian Road, Huichuan District, Zunyi, Guizhou China; 2 School of Nursing, Zunyi Medical University, Honghuagang District, Zunyi, Guizhou China; 3 Zheng ’an County People’s Hospital, Zheng’an, Guizhou China

**Keywords:** Sarco-osteoporosis, Osteosarcopenia, Middle-aged and elderly, Related factors, Nomogram

## Abstract

**Background:**

Sarco-osteoporosis is a skeletal muscle disease associated with aging and complex pathological factors. At present, there are few studies on the analysis of its related factors, and a nomogram to estimate the risk of sarco-osteoporosis in middle-aged and elderly patients is not available.

**Methods:**

A total of 386 patients admitted to our hospital from October 2021 to October 2022 were collected, and the general demographic data and clinical data of the patients were collected.386 subjects were enrolled in the study and randomly divided into training set and validation set at a ratio of 7:3. In the training set, the Least absolute shrinkage and selection operator(LASSO)regression technique was used to select the optimal predictive features, and multivariate logistic regression was used to screen the factors associated with sarco-osteoporosis, and a nomogram was constructed using meaningful variables from multivariate analysis. The performance of the nomograms was assessed and validated by Area Under Curve (AUC) and calibration curves.

**Results:**

There were no significant differences in baseline characteristic of individuals in training set and validation set, six variables with non-zero coefficients were screened based on LASSO regression in the training set. Multivariate logistic regression analysis showed that the related factors for sarco-osteoporosis in middle-aged and elderly inpatients included age (*OR* = 1.08, 95%*CI* 1.03 ∼ 1.14), regular exercise (*OR* = 0.29, 95%*CI* 0.15 ∼ 0.56), albumin (*OR* = 0.9, 95%*CI* 0.82 ∼ 0.98), height (*OR* = 0.93, 95%*CI* 0.88 ∼ 0.99) and lean mass index (*OR* = 0.66, 95%*CI* 0.52 ∼ 0.85), and a nomogram was constructed based on the above factors. AUC of nomogram were 0.868(95%*CI* 0.825 ∼ 0.912) in the training set and 0.737(95%*CI* 0.646 ∼ 0.828) in the validation set. Calibration curve analysis showed that the predicted probability of sarco-osteoporosis had high consistency with the actual probability, and the absolute error of the training set and verification set was 0.018 and 0.03, respectively.

**Conclusions:**

Our research showed that the occurrence of sarco-osteoporosis was associated with age, regular exercise, albumin, height and lean mass index, and we have developed a nomogram that can be effectively used in the preliminary and in-depth risk prediction of sarco-osteoporosis in middle-aged and elderly hospitalized patients.

## Background

Sarcopenia and osteoporosis is represent two chronic conditions which prevalence is increasing in the elderly population, both being recognized as a major health problem.

Sarcopenia is defined as an age-related loss of skeletal muscle mass plus loss of muscle strength and/or reduced physical performance, and it associated with an increased probability of adverse outcomes, such as falls, fractures, physical disability, and mortality [1]. The World Health Organization (WHO) defines osteoporosis as a disease characterized by low bone mass and microarchitectural deterioration of bone tissue, leading to enhance bone fragility and a consequent increase in fracture risk [2].Because the physiology and pathology of sarcopenia and osteoporosis overlap in many aspects, such as [3, 4] genetics, endocrine, hormones, nutrition and exercise, relevant experts suggest [5] that the simultaneous occurrence of both is defined as sarco-osteoporosis (SOP) or osteosarcopenia (OSP). The number of patients with SOP is increasing owing to the aging of society. Of course, SOP is not exclusive to the elderly, from the age of 50, the muscle mass of individuals decreases by 1–2% per year, and the muscle strength decreases by 1.5–3% per year between the ages of 50 and 60, and by 3% thereafter. The prevalence of SOP varies greatly among studies, a meta-analysis showed [6] that the prevalence of SOP in hospitalization and community environment was 5%∼40%, and the prevalence of high-risk groups such as falls or fractures was 27.2%∼40%. In addition to its high prevalence, SOP has a more serious burden on individuals and society than sarcopenia or osteoporosis alone, and is an important factor affecting the quality of life of patients [7, 8].

SOP is a common age-related disorder that often coexists with many chronic disease, such as cardiovascular disease, chronic obstructive pulmonary disease and diabetes [9, 10]. The presence of SOP will accelerate the occurrence and development of the above diseases and reduce the prognosis. Therefore, identifying SOP-related risk factors and early diagnosis are important for the treatment of these conditions. In addition, as a large group of chronic diseases, hospitalized patients should pay more attention to the occurrence of SOP. However, the current research on SOP mainly focuses on the pathological mechanism. Although there are some related factors studies, they mainly focus on postmenopausal women, the elderly and the community. There are few studies on the analysis of related factors for SOP in middle-aged and elderly inpatients, and the occurrence of SOP seriously affects the prognosis of hospitalized patients. Therefore, it is also necessary for early diagnosis of SOP in middle-aged and elderly inpatients.

To sum up, the purpose of this study is to analyze the related factors of SOP in middle-aged and elderly inpatients and develop a nomogram. Meanwhile, in order to help the researcher or clinical medical personnel is more convenient for middle-aged and elderly hospitalized patients with sarco-osteoporosis the risk of prediction, online version of the nomogram.

## Materials and methods

### Study Population

From October 2021 to October 2022 in Zunyi city, Guizhou Province, the spinal surgery inpatients were the overall study subjects. Inclusion criteria: (1) Age ≥ 50 years; (2) Ability to cooperate with relevant inspection, and volunteer to participate in research. Exclusion criteria: (1) Serious movement disorders of weight-bearing joint, movement disorder refers to the dysfunction of body regulation during voluntary movement, mainly manifested as limb tremor, limb stiffness, gait abnormality, etc.; (2) Neurological diseases affecting grip strength test; (3) History of hip or knee replacement; (4) Inflammatory musculoskeletal diseases; (5) Cognitive impairment, unable to cooperate with the relevant examination and questionnaire filling. The above exclusion criteria were excluded in this study because they would affect the accuracy of the outcome measures and the extrapolation of the prediction results.

The research follows the basic principles of the Declaration of Helsinki, the study protocol was approved by the Ethics committee of affiliated Hospital of Zunyi Medical University (No. KLLY-2021-132), and all individuals signed informed consent when participating in this study.

### Sample size

The calculation of sample size in this study was based on the principle of 10 times Events Per Variable, that is, each variable corresponds to 10 positive events [11]. In this study, 6 variables were involved in the multivariate analysis of the training set, that is, the minimum number of positive events was 60, and the final number of positive events was 102.

### Related definition

In this study, patients with the coexistence of sarcopenia and osteoporosis were defined as sarco-osteoporosis. Sarcopenia was defined using the diagnostic criteria of the Asian Working Group for Sarcopenia 2019 [1](AWGS 2019), related diagnostic thresholds are as follows: Low skeletal muscle mass is defined as Dual energy X-ray absorption (DXA), Male < 7.0 kg/m^2^, Female < 5.4 kg/m^2^; Low muscle strength is hand grip strength < 28 kg for Male and < 18 kg for Female [1]. Osteoporosis was defined according to the diagnostic criteria of WHO, osteoporosis as a T-score ≤-2.5 SD [12]. DXA was used to measure bone mineral density and skeletal muscle mass, and hand grip strength was measured with a grip meter. Geriatric nutritional risk index (GNRI) [13] = 1.489×albumin (g/L) + 41.7× (current weight/ideal weight). Ideal weight was calculated by the formula: Male ideal weight = height (cm) -100- [(height (cm)-150)/4]; Female ideal weight = height (cm) − 100 - [(height (cm) − 150) / 2. 5). NLR: neutrophil / lymphocyte ratio; PLR: platelet / lymphocyte ratio; LMR: lymphocyte / monocyte ratio; aggregate index of systemic inflammation (AISI): calculated by multiplying neutrophil count, monocyte count and platelet count and dividing the result by lymphocyte count. Systemic inflammatory response index (SIRI): Calculated by multiplying the neutrophil count and monocyte count and dividing the result by the lymphocyte count [14].

### Anthropometry and Body Composition

Anthropometric assessments by trained health assessors included body weight and height measured wearing light clothing without shoes All were measured to the nearest 0.1 unit and the mean of 2 measurements for weight and height was used. Body composition was measured using DXA and by physicians in the nuclear medicine department, including lean mass, fat mass, total body of Lean, total body of Fat, T-score, bone density of femoral neck, bone density of lumbar vertebrae and basal metabolic rate.

### Other Variables

The relevant variables selected in this study were prepared by the researchers on the basis of reading relevant literature [15–18]. Demographic data such as name, gender, age, smoking history, drinking history and fall history were collected by a self-designed general information questionnaire. Biochemical indicators such as albumin, total protein, hemoglobin and various inflammatory indicators were collected from the medical records. All data were collected by the researchers themselves, and patients with acute illness who were unable to cooperate with the relevant examinations and questionnaires were excluded before data collection, and all examinations and surveys were performed before the patients received various treatments.

### Statistical analysis

Due to the small amount of data in this study, in order to ensure the consistency of variable distribution between the training set and the validation set, the ratio of 7:3 was selected to divide the data into the training set and the validation set. A Shapiro-Wilk test was done to evaluated the normality of continuous variables. Normally distributed continuous variables were expressed as mean ± standard deviation (SD) and comparisons between two groups were made by two-tailed unpaired Student’s t-test. Non-normally distributed continuous variables were expressed by median (25% percentile, 75% percentile) and comparisons were made by a Mann–Whitney U-test. Categorical variables were shown as number (percentage) and were analyzed using the chi-square test. The logistic LASSO model was used to screen the predictive variables, the logistic LASSO model is a shrinkage method that can actively select from a large and potentially multicollinear set of variables in the regression, resulting in a more relevant and interpretable set of predictors. The selected variables were included in the multivariate logistic regression analysis. Risk factors that proved to be significant in the training set were used to create nomograms and the validity of the associated predictive factors was evaluated in the validation set. The calibration and discrimination of the nomogram were assessed using AUC and calibration curves, the Hosmer-Lemeshow test was used to evaluate the fit of the model. Statistical significance was assessed at a two-sided p value < 0.05. All analyses were conducted using R version 4.2.4.

## Results

### Characteristics of subjects

A total of 386 participants were divided into a training set and a validation set in a ratio of 7:3, the prevalence of SOP was 37.8% (102 subjects) in the training set and 34.5% (40 subjects) in the validation set, respectively. The characteristics of subjects are shown in Table 1. There were no significant differences in the characteristics of SOP status, gender, education level, residence, falls status, drinking, smoking, regular exercise, age, Number of comorbid diseases, height, weight, BMI, calcium, blood glucose, total protein, albumin, prealbumin, globulin, creatinine, GFR, ALT, AST/ALT, bilirubin, hemoglobin, neutrophils, lymphocytes, monocytes, platelets, white blood cells, NLR, PLR, LMR, AISI, SIRI, GNRI, calf circumference, hand grip strength, SLMI, total lean body mass, total body fat, FMI, LMI, BMR, T-score, bone femoral neck bone mineral density and lumbar spine bone mineral density between the two sets. Only the total hip bone mineral density (g/cm^2^) was different in the training set and the verification set, as shown in Table 1.

**Table 1:** Baseline characteristic of individuals in training set and validation set Mean ± SD/ [M(P_25_, P_75_)]/N(%)

Variables	Training set (N = 270)	Validation set (N = 116)	*P* values
SOP			0.617^b^
No	168(62.2%)	76(65.5%)	
Yes	102(37.8%)	40(34.5%)	
Gender			0.730^b^
Female	82(30.4%)	38(32.8%)	
Male	188(69.6%)	78(67.2%)	
Education level			0.667^b^
Below junior high school	196(72.6%)	81(69.8%)	
Junior high school and above	74(27.4%)	35(30.2%)	
Residence			0.766^b^
village	178(65.9%)	79(68.1%)	
urban	92(34.1%)	37(31.9%)	
Falls			0.349^b^
No	207(76.7%)	83(71.6%)	
Yes	63(23.3%)	33(28.4%)	
Drinking			0.103^b^
No	247(91.5%)	99(85.3%)	
Yes	23(8.5%)	17(14.7%)	
Smoking			0.229^b^
No	223(82.6%)	89(76.7%)	
Yes	47(17.4%)	27(23.3%)	
Regular exercise			0.574^b^
No	110(40.7%)	43(37.1%)	
Yes	160(59.3%)	73(62.9%)	
Age(year)	66.0[59.0–72.0]	64.0[56.0-72.2]	0.076^a^
Number of comorbid diseases	1.0[0.0–1.0]	1.0[0.0–1.0]	0.948^a^
Height(cm)	154.5[149.0-160.0]	154.0[150.0-158.2]	0.754^a^
Weight(kg)	57.0[51.0–63.0]	58.0[52.0–65.0]	0.269^a^
BMI (kg/m^2)	24.0[22.0-26.2]	24.3[22.2–26.9]	0.348^a^
Ca(mmol/L)	2.2[2.1–2.3]	2.2[2.1–2.3]	0.146^a^
Blood glucose (mmol/L)	5.0[4.6–5.5]	5.0[4.5–5.5]	0.710^a^
TP(g/L)	62.7[59.5–66.5]	62.0[59.2–66.2]	0.169^a^
Albumin (g/L)	38.6(4.0)	37.9(3.8)	0.101^a^
Globulin (g/L)	224.3[183.2–262.0]	210.5[180.8-255.8]	0.177^a^
Prealbumin (mg/L)	24.5[22.0–27.0]	24.0[22.0–26.0]	0.614^a^
Creatinine(umol/L)	67.5[59.0–81.0]	67.0[59.8–79.0]	0.722^a^
GFR (ml(min1.73 m^2))	89.4[78.2-105.8]	92.2[80.8-103.8]	0.412^a^
ALT(U/L)	18.0[13.0-27.8]	18.0[13.8–26.5]	0.725^a^
AST(U/L)	23.5[19.0–30.0]	23.0[19.0-29.2]	0.679^a^
AST/ALT	1.3[1.0-1.7]	1.3[1.1–1.6]	0.521^a^
Bilirubin(umol/L)	10.6[8.2–14.3]	10.1[8.0-13.5]	0.391^a^
Hb(g/L)	125.5(17.0)	124.1(21.0)	0.516^a^
Neutrophile (10^9/L)	3.8[2.7–5.3]	3.6[2.9–4.6]	0.343^a^
Lymphocyte (10^9/L)	1.5[1.2–1.9]	1.7[1.2-2.0]	0.261^a^
Monocyte (10^9/L)	0.5[0.4–0.6]	0.5[0.4–0.6]	0.837^a^
Blood platelet (10^9/L)	206.5[164.0-249.0]	218.0[178.8-261.5]	0.088^a^
WBC (10^9/L)	6.2[4.9–7.8]	6.1[5.0-7.2]	0.650^a^
NLR	2.4[1.6–3.8]	2.1[1.5–3.3]	0.110^a^
PLR	129.0[97.8–177.0]	132.0[101.6-177.6]	0.608^a^
LMR	3.3[2.3–4.3]	3.6[2.4–4.8]	0.159^a^
AISI	236.0[131.8-443.5]	213.1[146.9-379.9]	0.617^a^
SIRI	1.1[0.7–2.2]	1.0[0.7–1.6]	0.199^a^
GNRI	102.7(9.0)	102.5(9.2)	0.859a
CC (cm)	32.3[31.0–34.0]	33.0[31.1–34.0]	0.186^a^
HGS (kg)	19.5[16.0-24.6]	20.1[15.9–25.4]	0.844^a^
SLMI (kg/m^2)	5.4[4.8-6.0]	5.6[5.2–6.1]	0.052^a^
Total body of Lean	55.2[49.8–60.0]	53.6[49.9–60.8]	0.904^a^
Total body of Fat	42.4[37.5–47.9]	43.8[35.9–47.7]	0.926^a^
FMI (kg/m^2)	10.4[8.4–12.3]	10.9[8.2–13.0]	0.557^a^
LMI (kg/m^2)	13.4[12.3–14.6]	13.6[12.6–14.7]	0.307^a^
BMR (kcal/Day)	1188.6[1108.1-1290.5]	1214.4[1114.0-1305.9]	0.122^a^
T-score	-3.2[-4.0;-2.5]	-2.9[-4.1;-2.2]	0.426^a^
Neck BMD(g/cm2)	0.6[0.6–0.7]	0.6[0.6–0.7]	0.189^a^
Total hip BMD(g/cm2)	0.7 ± 0.1	0.8 ± 0.1	0.040^c^
L1BMD(g/cm2)	0.6[0.5–0.7]	0.6[0.5–0.7]	0.498^a^
L2BMD(g/cm2)	0.6[0.5–0.7]	0.6[0.5–0.7]	0.239^a^
L3BMD(g/cm2)	0.7[0.6–0.8]	0.7[0.6–0.8]	0.617^a^
L4BMD(g/cm2)	0.7[0.6–0.8]	0.7[0.6–0.8]	0.671^a^
TLBMD	0.6[0.6–0.7]	0.7[0.5–0.8]	0.437^a^

a: Mann-Whitney test; b: chi-square test; c: t test; SOP: sarco-osteoporosis; BMI: body mass index; AST: aspartate aminotransferase; GFR: glomerular filtration rate; ALT: alanine aminotransferase; NLR: neutrophil/lymphocyte ; PLR: platelet/lymphocyte; LMR: lymphocyte/monocyte; AISI: systemic inflammation aggregation index; SIRI: Systemic inflammatory response index; GNRI: geriatric nutritional risk index; SMMI: skeletal muscle mass index; FMI: fat mass index; LMI: lean mass index; BMR: basal metabolic rate; BMD: Bone mineral density

### Variable selection and multivariate analysis of SOP

**Fig. 1:**
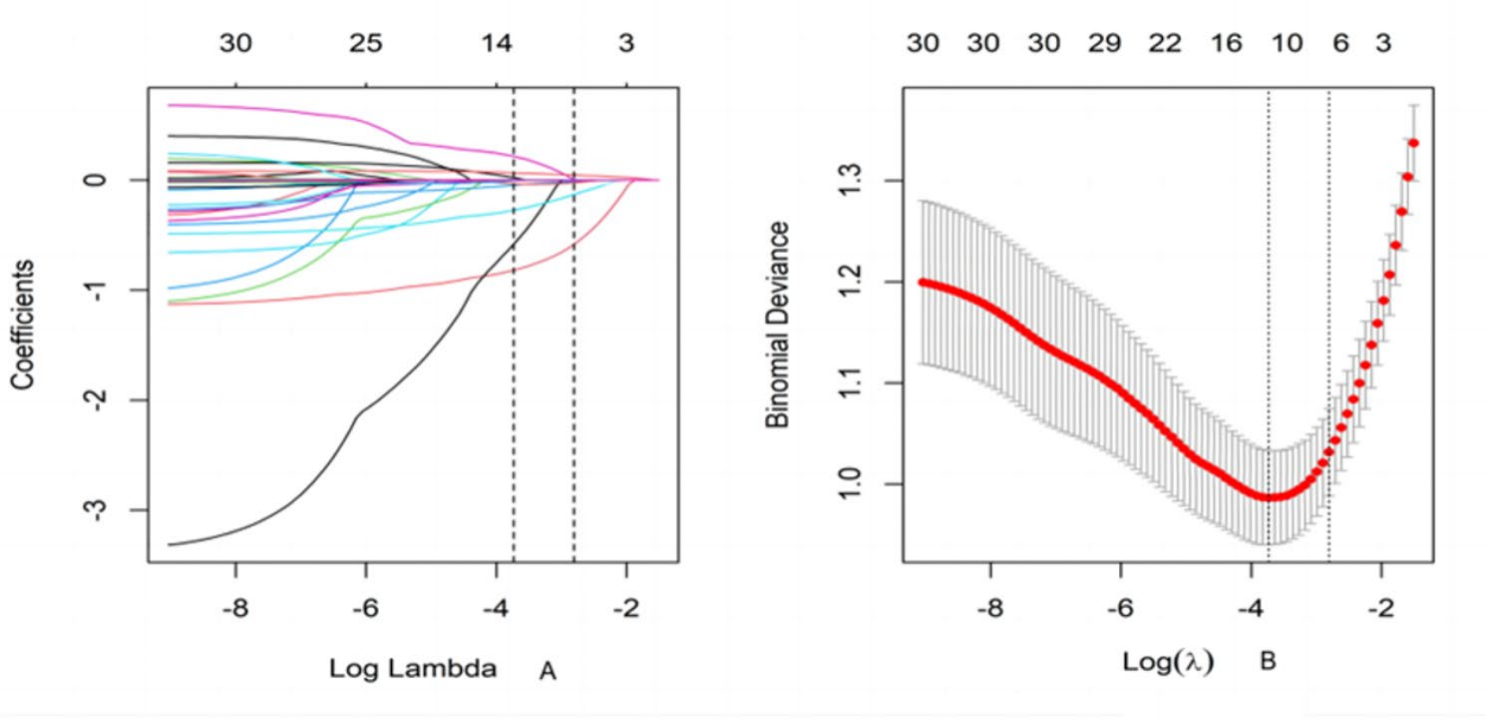
Variable selection plots for SOP

In the training set, the subjects were divided into two groups according to whether SOP occurred. The variables of the two groups were compared between the two groups, and the variables with differences were selected for LASSO regression to further screen the variables. Six variables with non-zero coefficients were selected by LASSO regression, these variables included Age, Regular exercise, Height, Albumin, LMI and BMR (Fig. 1). Multivariate regression analysis was conducted with whether SOP occurred as the dependent variable, and the results showed that that age, regular exercise, height, albumin and LMI were independent risk factors for SOP (Table 2).

**Table 2:** Binary logistic multivariate analysis of SOP

Items	Unadjusted *OR* (95%*CI*)	Adjusted *OR* (95%CI)	*P value*
Age(year)	1.14(1.1–1.19)	1.08(1.03–1.14)	**0.003**
Regular exercise			
No	Reference		
Yes	0.22(0.13–0.38)	0.29(0.15–0.56)	**< 0.001**
Height(cm)	0.91(0.88–0.94)	0.93(0.88–0.99)	**0.013**
Albumin (g/L)	0.84(0.78–0.91)	0.9(0.82–0.98)	**0.015**
LMI (kg/m^2)	0.59(0.49–0.71)	0.66(0.52–0.85)	**0.001**
BMR (kcal/Day)	0.9903(0.9875–0.9931)	0.9975(0.9933–1.0017)	0.241

Bold represent *P* < 0.05; LMI: Lean mass/height^2^; BMR: Basal metabolic rate

### Construction of sarco-osteoporosis nomogram model

**Fig. 2:**
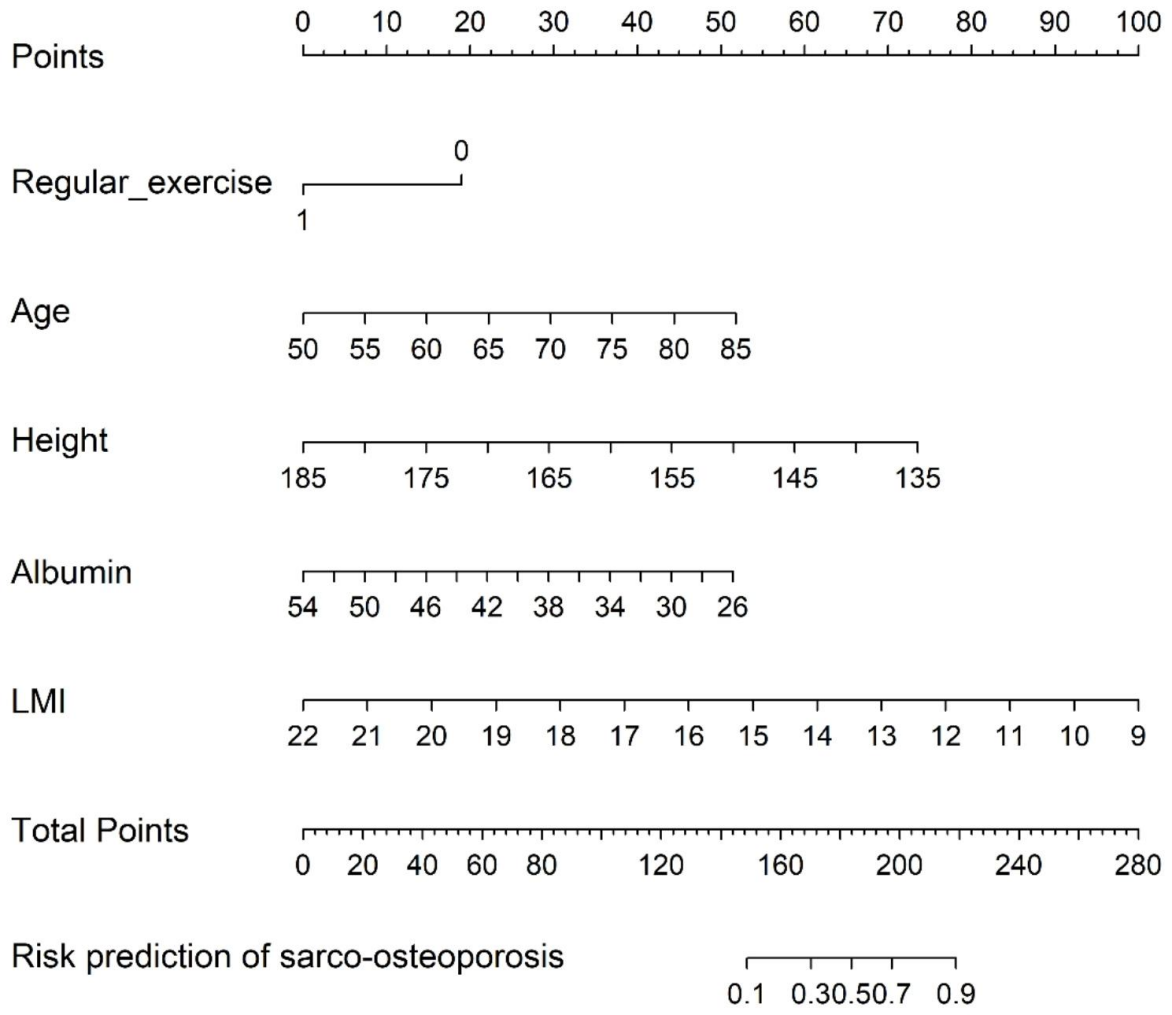
The nomogram for the prediction of sarco-osteoporosis. The points of each features were added to obtain the total points, and a vertical line was drawn on the total points to obtain the corresponding ‘risk of sarco-osteoporosis’, LMI: Lean mass/height^2^

This study developed a nomogram using the analysis of meaningful variables in the training set (Fig. 2): Regular exercise, Age, Height, Albumin, and LMI. The score corresponding to the value of each prediction variable is calculated by the column chart, and the total score is obtained by adding up the score of each prediction variable. In order to help the researcher or clinical medical personnel is more convenient for middle-aged and elderly hospitalized patients with sarco-osteoporosis the risk of prediction, online version of the nomogram (available through https://anananan1.shinyapps.io/OPSP/).

### Assessment of the nomogram of sarco-osteoporosis

In this study, the AUC and calibration curves were used to evaluate the nomogram.

**Fig. 3:**
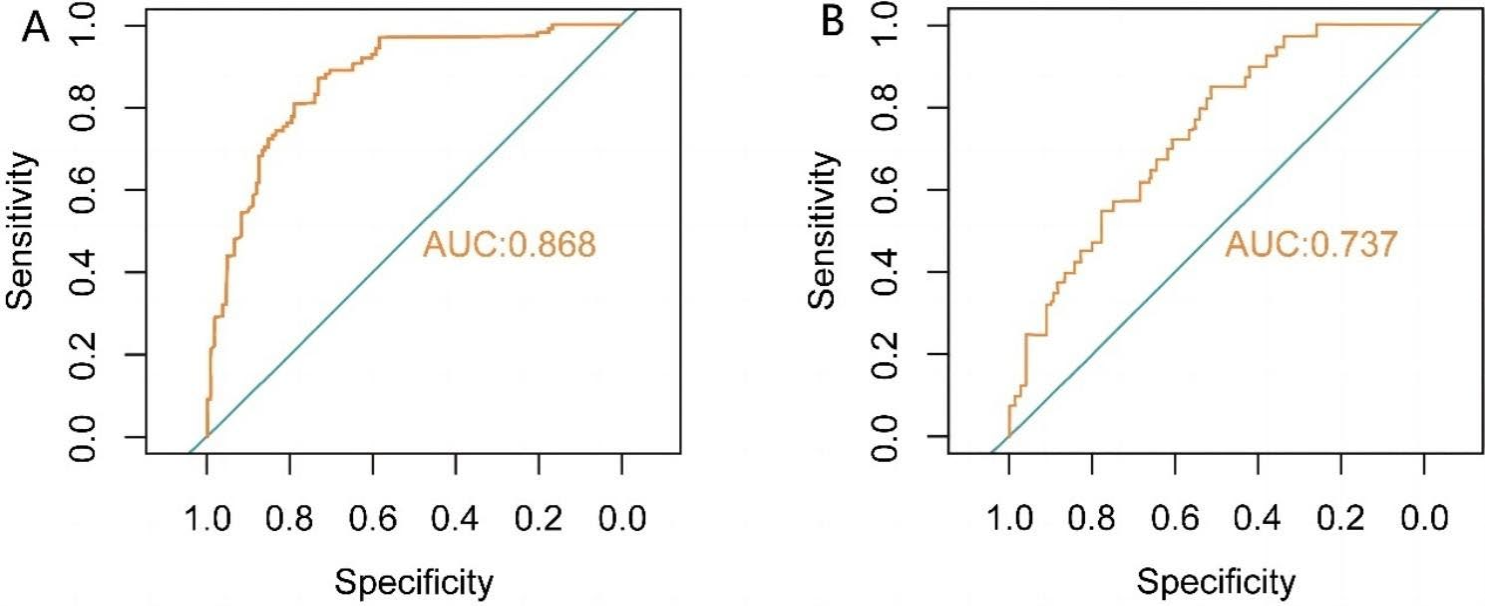
Receiver operating characteristic (ROC) curves for the prediction of SOP in the training set and validation set. **(A)** ROC curves of nomogram in the training set. **(B)** ROC curves of nomogram in the validation set.

**Fig. 4:**
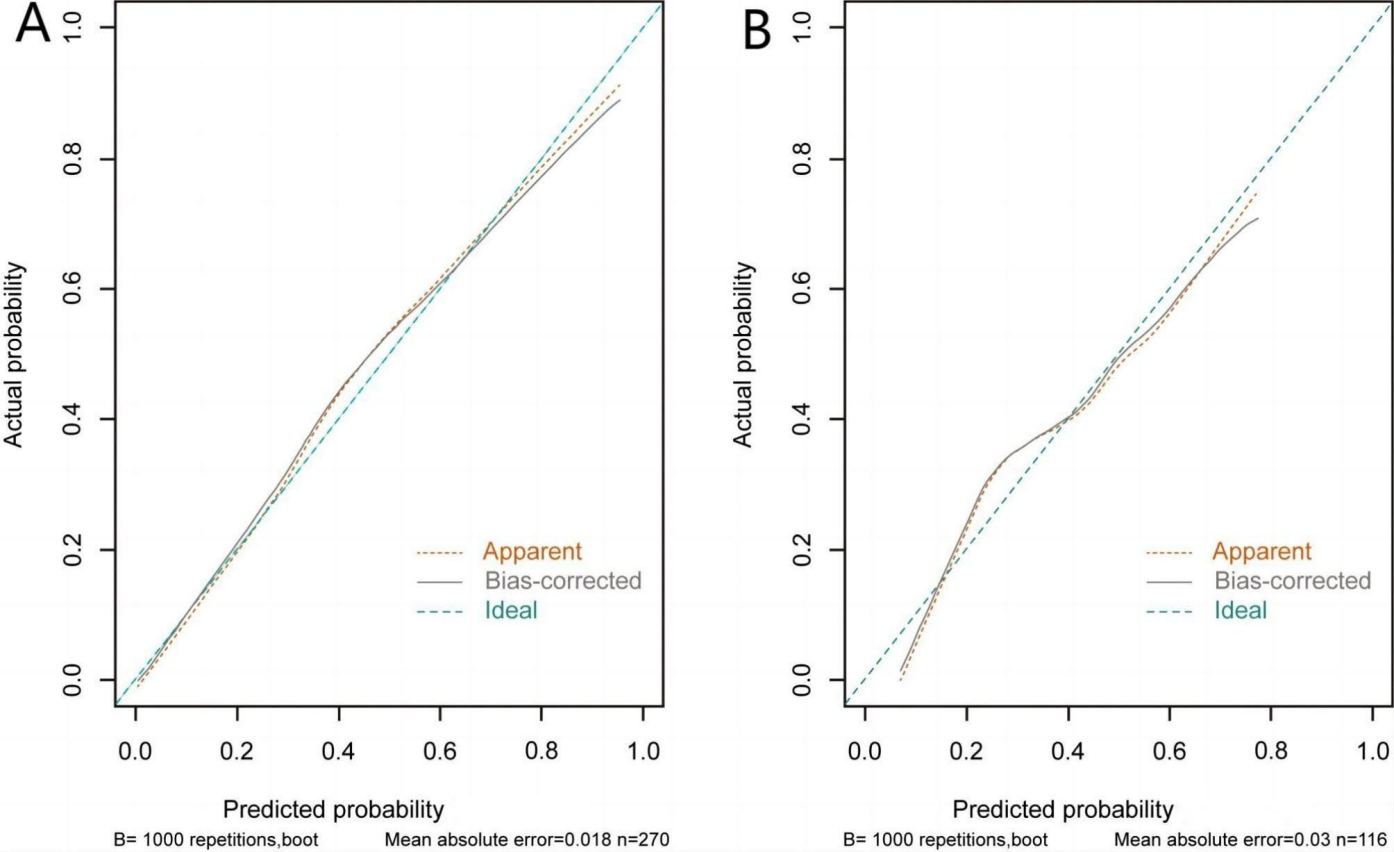
Calibration curves of the nomogram prediction in the training set and validation set. **(A)** Calibration curves of nomogram in the training set. **(B)** Calibration curves of nomogram in the validation set.

The *P* value of Hosmer-Lemeshow test was greater than 0.05 in both the training set and the validation set, indicating the model fits well. The AUC values of the training set and the validation set were 0.868(95%*CI* 0.825 ∼ 0.912) and 0.737(95%*CI* 0.646 ∼ 0.828) respectively, manifesting that the nomogram model based on the training set had well discrimination, and the model effect was better in the training set (Fig. 3). And the calibration curve showed that the predicted probability of sarco-osteoporosis had a high consistency with the actual probability. The absolute error in the training set and the validation set were 0.018 and 0.03, respectively, indicating that the model was well calibrated (Fig. 4).

## Discussion

Because of the serious impact of SOP on individual health, especially for hospitalized patients, exploring its related factors and timely diagnosis is crucial for effective treatment. Therefore, it is necessary to establish a predictive model to estimate the risk of SOP in order to implement effective management. In this study, non-zero coefficient variables were screened by LASSO regression, and the factors related to SOP were analyzed by multivariate logistic regression. The results showed that the variables related to SOP were age, height, LMI, regular exercise and albumin. The nomogram constructed based on the above variables also has moderate prediction performance.

The occurrence of SOP is related to the interaction of many factors, and age is one of the important factors. It is well known that the components of the musculoskeletal system change with age, such as decreased bone mineral density and number of muscle fibers, loss of muscle strength and muscle mass [19]. The main causes of the above composition changes include hormonal changes, increased oxidative stress, poor nutritional status and lack of activity [20, 21]。In other words, the above reasons lead to the decrease of skeletal muscle metabolic signals and increase the prevalence of SOP.

The effect of height on SOP is rarely reported. Our study showed that height is associated with SOP (*OR* = 0.93; 95%*CI*: 0.88 ∼ 0.99). This finding in our study may be due to the fact that some participants had an osteoporotic fracture before hospitalization, which resulted in a significantly lower height in the SOP group than in the non-SOP group. In a longitudinal study [22], height loss of more than 4 cm was a risk factor for falls (*OR* = 2.676, 95%*CI*: 1.122 ∼ 6.284) and sarcopenia (*OR* = 2.676, 95%*CI*: 1.122 ∼ 6.284). A survey on the status of osteoporosis in men aged 50 and over in Taiwan by Ko et al [23]. showed that height (*OR* = 0.94, 95%*CI*: 0.92 ∼ 0.95) was associated with osteoporosis. As mentioned in the background, muscle and bone are interconnected, so it is possible that changes in bone cause changes in height, resulting in lower height in SOP patients. However, this study was found in hospitalized patients. Due to the high incidence of sarcopenia in patients with osteoporosis, whether height is simply related to osteoporosis or sarcopenia or related to SOP cannot be accurately answered.

Changes in muscle mass also play an important role in the development of SOP. In general, the higher the lean mass index (LMI), the higher the muscle mass. LMI as one of the evaluation parameters of SOP diagnosis, our research showed that the higher the LMI, the lower the prevalence of SOP (*OR* = 0.66, 95%*CI*: 0.52–0.85, *P* < 0.001). Previous studies have shown [24]that the correlation of skeletal muscle is also reflected in the change of one of the components will also cause the change of other components. Patients with sarcopenia had lower bone mineral density, and the occurrence of sarcopenia increased the risk of OP (*OR* = 7.3, *P* < 0.001) [25]. In the case of independent of other risk factors, a previous decrease in lean body mass (rather than fat mass) was associated with an increased risk of fractures, especially hip fractures. The risk of hip fractures increased by 29–38% for every standard deviation loss of lean body mass [26]. The above studies all indicate that bone and muscle are two closely related components of the human body. Therefore, the prevalence of SOP in patients with low LMI will also increase correspondingly.

It was also found that regular exercise habits and albumin content were correlated with SOP. Sipild et al [27]. showed that the level of physical activity in perimenopausal women was related to appendiceal skeletal muscle mass (β = 0.278, 95% CI: 0.179 ∼ 0.37) and femoral neck BMD (β = 0.227, 95%*CI* 0.097 ∼ 0.356). Fahimfar et al. [28] reported a significant negative correlation between physical activity and sarco-osteoporosis in men (PR = 0.64, 95%*CI* 0.46 ∼ 0.88), but not in women. Related studies also showed that protein intake was associated with sarco-osteoporosis, compared with non-sarco-osteoporosis patients, sarco-osteoporosis patients had lower total protein intake (*P* < 0.001) [29]. There is also research evidence that dietary therapy plays an indispensable role in the prevention and treatment of sarco-osteoporosis, and in elderly individuals, it is recommended that the diet should provide at least 1.0-1.2 g/(kg/d) of protein [30]. Exercise and nutrition are effective stimuli for muscle protein synthesis, which can effectively activate bone metabolism and muscle metabolism pathways, and reduce oxidative stress and inflammatory factors [31]. Therefore, relevant research data support the view that age-related skeletal muscle function decline can also be affected by lifestyle factors (such as diet and exercise), that is, the same population may have different body composition due to different lifestyles [32, 33]. Currently, pharmaceutical treatments for SOP are currently unavailable, So it is very important to address modifiable factors to prevent, or at least delay, the onset of SOP [34].

In this study, age, height, lean mass index, regular exercise and albumin were included in the nomogram model by LASSO regression and multivariate logistic regression analysis, we followed the recommendations of the Multivariable Prediction Model for Transparent Reporting of Individual Prognosis or Diagnosis (TRIPOD) statement [35], used the Bootstrap method for internal validation, and evaluated the performance of the model by AUC and calibration curve. The results showed that the AUC values of the model constructed by the relevant variables in the training set and the validation set were 0.868 (95%*CI* 0.825 ∼ 0.912) and 0.737(95%*CI* 0.646 ∼ 0.828), respectively, suggesting that the model had good discrimination and performed better in the training set. The Hosmer-Lemeshow goodness of fit test showed that the model fitted well, and then the Bootstrap method was used for internal verification of the model, indicating that the model had good calibration. Since no SOP-related prediction model has been reported, the conclusions of this study cannot be compared with other studies. In addition, because the nomogram constructed to calculate the risk of SOP is still cumbersome, this study also provides a dynamic version of the web page of the nomogram, which can quickly obtain the risk of SOP, and has certain promotion significance and clinical application value. Of course, the risk factors of SOP varied widely across studies. Age, Gender, Nutrition, and daily activities status were the most common independent factors in the studied population, and apart of them were included in our prediction models. However, there is no agreement among investigators as to what constitutes a major predictor. It is therefore suggested that a SOP risk prediction model developed in the particular racial, ethnic, or national groups may not be directly applied to other populations.

There are also some limitations in our study. Firstly, as mentioned above, the diagnosis of sarcopenia we only measure muscle mass and strength, not body function, which may result in a bias of missed diagnosis. Secondly, the nomogram showed medium prediction accuracy may suggest that other factors should be included. Thirdly, this study is a single-center cross-sectional study involving only 386 samples, and the selected variables may not be comprehensive. And there may be measurement errors during measurement, which mainly come from the accurate performance of the instrument and the measurement method. These may have inevitably caused bias. The prediction accuracy could perhaps be improved in further studies with large sample sizes and more variables. Further multicenter external validation should be performed to verify the discriminating ability and generalizability of our nomogram.

## Conclusion

In conclusion, based on a cross-sectional study, we analyzed the related factors of sarco-osteoporosis, which included age, regular exercise, height, albumin and LMI, and we developed and validated a simple nomogram to predict the risk of SOP for the middle-aged and elderly hospitalized patients. The nomogram demonstrated a degree of predictive accuracy and discrimination in the training set and validation set. This visualization model and website will aid the patients and physicians to predict the risk of SOP and better clinical management.

## Data Availability

The datasets generated and/or analyzed during the current study are not publicly available due data sharing was not included in the informed consent process, but are available from the corresponding author on reasonable request.

## Abbreviations

WHO: World Health Organization
LASSO: Least absolute shrinkage and selection operator
AUC: Area Under Curve
SOP: Sarco-osteoporosis
AWGS 2019: Asian Working Group for Sarcopenia 2019
DXA: Dual energy X-ray absorption
GNRI: Geriatric nutritional risk index
AISI: Aggregate index of systemic inflammation
SIRI: Systemic inflammatory response index
LMI: Lean mass index
